# The Variation and Correlation of Serum Adiponectin, Nesfatin-1, IL-6, and TNF-α Levels in Prediabetes

**DOI:** 10.3389/fendo.2022.774272

**Published:** 2022-03-03

**Authors:** Kangkang Huang, Yunlai Liang, Yating Ma, Jiahui Wu, Huidan Luo, Bin Yi

**Affiliations:** ^1^ Department of Clinical Laboratory, Xiangya Hospital, Central South University, Changsha, China; ^2^ National Clinical Research Center for Geriatric Disorders, Xiangya Hospital, Central South University, Changsha, China

**Keywords:** T2DM, prediabetes, adiponectin, nesfatin-1, TNF-α, IL-6

## Abstract

**Background:**

The variation and correlation among adiponectin, nesfatin-1, tumor necrosis factor α (TNF-α), and interleukin 6 (IL-6), which may be involved in the development of the decline of health into prediabetes and diabetes, have not been elucidated. This study aims to investigate the roles of these cytokines in this process.

**Methods:**

Seventy-two type 2 diabetes mellitus (T2DM) patients, 75 prediabetics, and 72 healthy individuals were enrolled in our case control study. Serum adiponectin, nesfatin-1, TNF-α, and IL-6 were tested with appropriate kits, and primary data were analyzed with correct methods.

**Results:**

Serum levels of each cytokine in patients with prediabetes were between T2DM and the healthy, and significant differences were found among them. TNF-α and nesfatin-1 levels in T2DM were obviously different compared to prediabetes or the healthy; IL-6 and adiponectin levels in the healthy group were significantly changed in contrast to prediabetes or T2DM. Correlation analysis found that in prediabetics, adiponectin was positively correlated with TNF-α (R = 0.2939, *P* = 0.0105) and IL-6 (R = 0.3918, *P* = 0.0005), and their relationship was greatly strengthened in prediabetes accompanied by insulin resistance (TNF-α: R = 0.7732, *P* < 0.0001, IL-6: R = 0.6663, *P* = 0.0005). We also demonstrated that declined adiponectin (OR = 6.238, *P* = 0.019) and nesfatin-1 (OR = 2.812, *P* = 0.01) and elevated TNF-α (OR = 5.541, *P* = 0.001) were risk factors for prediabetes toward diabetes.

**Conclusions:**

This research proved significant variations of adiponectin, nesfatin-1, IL-6, and TNF-α levels in the healthy, prediabetics, and T2DM, suggesting a slow and gradual change during the progression from a healthy condition toward diabetes *via* prediabetes.

## Background

Prediabetes occurs before type 2 diabetes mellitus (T2DM) and is a high-risk state for diabetes development. It is predicted that there will be 470 million prediabetes by 2030 (1). Until 2019, there were nearly 463 million T2DM patients, and this number will increase to 700 million in 2045 (2). T2DM is characterized by insulin resistance and islet β-cell dysfunction. Some studies believed that β-cell dysfunction started even before the impaired glucose tolerance (3). It has been proven that inflammation in islet of T2DM patients is due to immune cell infiltration (4), amyloid deposition (5), cell death (6), and fibrosis (7). Tumor necrosis factor α (TNF-α) and interleukin 6 (IL-6) as pro-inflammatory cytokines secreted by M1 macrophage (8) could impair insulin secretion through degrading insulin receptor substrate (9). Elevated TNF-α and IL-6 levels in comparison to healthy controls were observed in T2DM serum (10). However, the variation of TNF-α and IL-6 levels in prediabetes and their comparison with T2DM and the healthy group have not been disclosed.

Adiponectin is secreted by adipocytes, first described as a protein similar to complement 1q (11). Adiponectin is a 30-kDa monomeric glycoprotein (12), which exerts extensive physiological effects such as improving insulin sensitivity (13), promoting anti-inflammatory (14), and enhancing vascular protection (15). Several case-control studies (16, 17) and meta-analysis (18) proved that decreased circulating adiponectin in serum was associated with high T2DM risk. Nesfatin-1 is a satiety molecule found in the hypothalamus by Oh-I et al. (19) in 2006. Nesfatin-1 was found to be able to improve insulin resistance and glucose uptake through Akt/Adenosine Monophosphate activated Protein Kinase (AMPK)/Target of Rapamycin Complex 2 (TORC2) pathway in an animal study (20). Recently, some studies on the role of nesfatin-1 in the development of T2DM confirmed the variation of serum nesfatin-1 levels in T2DM, but their conclusions were not consistent (21, 22). A meta-analysis figured out that serum nesfatin-1 level elevated in newly diagnosed T2DM but decreased in those patients who received antidiabetic treatment (23). However, few studies have focused on the change of nesfatin-1 levels in prediabetes (24, 25).

So far, the variation of adiponectin, nesfatin-1, IL-6, and TNF-α levels in T2DM has been extensively explored, which identified elevated levels of IL-6 and TNF-α and reduction of adiponectin and nesfatin-1 levels. However, the change of these markers in prediabetes and their correlation have not been delineated. In this case control study, we aimed to probe into the differences and connections of adiponectin, nesfatin-1, IL-6, and TNF-α levels in prediabetics, T2DM, and the normal participants in order to better understand the roles of these cytokines in the development of prediabetes and T2DM.

## Materials and Methods

From August 2020 to February 2021, 75 prediabetics (58.56 ± 9.73years) and 72 T2DM (58.07 ± 9.60 years) consecutive participants who attended Xiangya Hospital of Central South University (Hunan province, China) and 72 (56.88 ± 6.59 years) age- and sex-matched healthy volunteers with normal glucose metabolism were introduced in this case control study. According to the criteria of American Diabetes Association (26), criteria for T2DM include fasting blood glucose (FBG) ≥126 mg/dl (7.0 mmol/L) or 2-h blood glucose (2-h BG) ≥200 mg/dl (11.1 mmol/L) during oral glucose tolerance test (OGTT) or HbA1c ≥6.5% (48 mmol/mol) or a random plasma glucose ≥200 mg/dl (11.1 mmol/L) for patients with classic symptoms of hyperglycemia or hyperglycemic crisis, and the criteria required for prediabetes include FBG: 100~125 mg/dl (5.6~6.9 mmol/L) or 2-h BG during 75-g OGTT: 140~199 mg/dl (7.8~11.0 mmol/L) or HbA1c: 5.7%~6.4% (39–57 mmol/mol). Patients with hypertension, liver disease, heart disease, renal disease, cancer, or other chronic diseases as well as pregnant women were excluded. All participants were given informed consent, and this study was permitted by the ethics committee of Xiangya Hospital of Central South University (No. 202009119).

In this study, 5 ml venous blood was extracted for biochemical analysis from each participant who fasted at least 8 h. Serum was collected by centrifuging the blood at 3,600 rpm for 5 min and stored at -20°C until detection. OGTT was conducted for 2-h BG. All tests were performed at Xiangya Hospital of Central South University. Serum levels of nesfatin-1, IL-6, and TNF-α were determined by enzyme-linked immunosorbent assay (ELISA) kit (Jiangsu Meimian Industrial Co., Ltd, Jiangsu, China), while adiponectin was measured by latex enhanced immune turbidimetry (Guangdong Uniten Biotechnology, Guangdong, China) on AU5800 chemistry analyzer (Beckman Coulter, CA, USA). These kits show negligible cross-reactivity (< 0.5%) and high sensitivity (detection limit of 0.5 µg/ml for adiponectin, 30 pg/ml for nesftain-1, 0.8 ng/ml for IL-6, and 20 ng/ml for TNF-α). Insulin was detected with chemiluminescent immunoassay (Beckman Coulter, CA, USA). Other parameters such as total triglyceride (TG), total cholesterol (TC), high-density lipoprotein cholesterol (HDL-C), low-density lipoprotein cholesterol (LDL-C), total bilirubin (TB), direct bilirubin (DB), alanine aminotransferase (ALT), aspartate aminotransferase (AST), total bile acid (TBA), serum creatinine (Scr), FBG, and 2-h BG were detected on AU5800 chemistry analyzer (Beckman Coulter, CA, USA) with the appropriate kits. Hemoglobin A1c (HbA1c) was quantified using high-performance liquid chromatography (ARKRAY, Kyoto, Japan). Indexes related to insulin were calculated according to the following formulas: Homeostasis model assessment of insulin resistance (HOMA-IR) = insulin (µU/ml) _*_ FBG (mmol/l)/22.5; homeostasis model assessment of beta-cell function (HOMA-β) = 20 _*_ insulin (μU/ml)/[FBG (mmol/L)-3.5]; homeostasis model assessment of insulin sensitivity (HOMA-IS) = 22.5/insulin (μU/ml) _*_ FBG (mmol/l) (27); and estimated glomerular filtration rate (eGFR) (MDRD) [ml·min^-1^·(1.73 m^2^)^-1^] =30,849 _*_ [Scr (µmol/L)]^-1.154^_*_ (age)^-0.203^ (multiply by 0.742 for women) (28).

### Statistical Analysis

Statistical analysis was carried out using SPSS version 26 for Windows (SPSS Inc., IL, USA). The results were presented as mean ± standard deviation for continuous variables and as numbers for categorical variables. Differences among groups or subgroups were calculated with ANOVA, and differences between groups were calculated with SNK test. Correlation between nesfatin-1 and other indexes was analyzed with Pearson correlation test. Influence factors involving the process of developing prediabetes into T2DM or interfering with HOMA-IR and HbA1c were determined by logistic regression analysis. *P* < 0.05 (two-tailed) was regarded as statistically significant.

## Results

### General Characteristics

**Figure 1:**
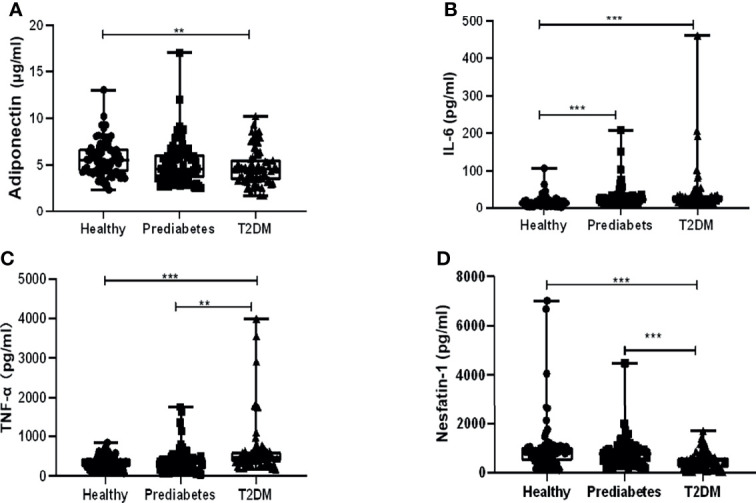
Comparison of serum levels of adiponectin, interleukin 6 (IL-6), tumor necrosis factor α (TNF-α), and nesfatin-1 among type 2 diabetes mellitus (T2DM), prediabetes, and the healthy. **(A)** Adiponectin levels in T2DM significantly decreased compared with those in the healthy other than prediabetes, while no apparent difference was observed between T2DM and prediabetes. **(B)** IL-6 levels in T2DM or prediabetes were dramatically increased in contrast to those in the healthy, while no significant variation was displayed between the prediabetes and T2DM. **(C)** Profound elevation of TNF-α in T2DM was found compared with prediabetes or the healthy group, but it showed an insignificant rise when comparing prediabetes with healthy. **(D)** Nesfatin-1 levels in T2DM were dramatically declined in contrast to those in the healthy or prediabetes, while no significant reduction was presented when comparing prediabetes with the healthy. ** vs. Healthy *P* < 0.01, *** vs. Healthy *P* < 0.001.

In total, 219 participants were enrolled in this case control study, and their anthropometric characteristics and clinical parameters were listed in **Table 1**. There were no noteworthy differences in gender composition, age, and body mass index (BMI) among the three groups. Results showed their concentrations in prediabetes vs. in T2DM vs. in the healthy to be as follows: adiponectin (5.12 ± 2.31 vs. 4.79 ± 1.92 vs. 5.74 ± 1.91 µg/ml), nesfatin-1 (802.58 ± 570.13 vs. 489.85 ± 349.92 vs. 1,034.54 ± 1,174.07 pg/ml), TNF-α (382.81 ± 308.63 vs. 663.95 ± 693.19 vs. 342.06 ± 160.89 pg/ml), and IL-6 (29.69 ± 29.41 vs. 38.46 ± 60.48 vs. 16.55 ± 15.29 pg/ml), indicating that the serum levels of these cytokines in prediabetes were between those in T2DM and the healthy group. Variations of the levels among them were statistically analyzed. As shown in **Figure 1A**, serum adiponectin levels in T2DM or prediabetes were significantly decreased compared with those in the healthy, while no apparent difference was observed between T2DM and prediabetes. Serum IL-6 levels in T2DM or prediabetes were dramatically increased in contrast to the healthy, while no significant variation was displayed between the prediabetes and T2DM (**Figure 1B**). Profound elevation of TNF-α in T2DM was found compared with prediabetes or the healthy group, but it showed an insignificant rise when comparing prediabetes with the healthy (**Figure 1C**). Nesfatin-1 levels in T2DM were dramatically declined in contrast to those in the healthy or prediabetes, while no significant reduction was presented when comparing prediabetes with the healthy (**Figure 1D**).

**Table 1:** Anthropometric and clinical characteristics of the study subjects in different groups.

Parameter	T2DM	Prediabetes	Healthy
Male/female	50/29	49/26	47/25
Age (years)	58.07 ± 9.60	58.56 ± 9.73	56.88 ± 6.59
BMI (kg/m^2^)	22.88 ± 3.85	22.17 ± 2.68	22.93 ± 2.46
IL-6 (pg/ml)	38.46 ± 22.23	29.69 ± 29.41^*^	16.55 ± 15.29^#^
TNF-α (pg/ml)	663.95 ± 693.19^**#^	382.81 ± 308.63	342.06 ± 160.89
Nesfatin-1 (pg/ml)	489.85 ± 349.92^**#^	802.58 ± 570.13	1,034.54 ± 1,174.07
Adiponectin (μg/ml)	4.79 ± 1.92^*^	5.12 ± 2.31	5.71 ± 1.91
Insulin (μU/ml)	12.16 ± 10.02^*^	11.14 ± 12.14^*^	5.89 ± 2.77^#^
HOMA-IR	4.85 ± 5.57^**#^	3.19 ± 3.52^**^	1.37 ± 0.70^##^
HOMA-β	66.05 ± 79.55	76.43 ± 81.45	68.57 ± 40.71
HOMA-IS	0.44 ± 0.67^**^	0.58 ± 0.40^**^	0.93 ± 0.56^##^
TG (μmol/L)	2.31 ± 2.73^*^	2.14 ± 1.83^*^	1.25 ± 0.41^#^
TC (μmol/L)	4.84 ± 0.73^#^	5.52 ± 1.05^*^	5.11 ± 1.63^#^
LDL-C (mmol/L)	3.21 ± 0.99^#^	3.50 ± 0.71^*^	2.99 ± 0.55^#^
HDL-C (mmol/L)	1.14 ± 0.32^*#^	1.28 ± 0.30	1.35 ± 0.26
HDL/LDL	0.42 ± 0.48	0.38 ± 0.14	0.46 ± 0.11
TB (μmol/L)	11.43 ± 5.35	11.89 ± 6.09	10.83 ± 3.19
DB (μmol/L)	5.58 ± 2.72	5.76 ± 2.84	5.45 ± 1.55
ALT (U/L)	27.95 ± 32.12	31.39 ± 24.52^*^	21.77 ± 8.75^#^
AST (U/L)	25.44 ± 16.71	27.87 ± 12.22	23.83 ± 5.46
TBA (μmol/L)	5.03 ± 5.12	4.62 ± 3.78	3.64 ± 2.67
Scr (μmol/L)	100.67 ± 70.87^*#^	79.94 ± 13.16	74.83 ± 12.49
eGFR [ml·min^-1^ (1.73 m^2^)^-1^]	74.55 ± 23.20	80.93 ± 26.13	79.43 ± 9.69
HbA1c (%)	7.81 ± 1.94^*#^	5.95 ± 0.26^*^	5.49 ± 0.35^#^
FPG (mmol/l)	8.78 ± 3.14^*#^	6.41 ± 0.34^*^	5.28 ± 0.43^#^
2-h BG (mmol/l)	14.85 ± 5.40^**#^	9.06 ± 1.38	5.99 ± 0.95

T2DM, type 2 diabetes mellitus; BMI, body mass index; IL-6, interleukin 6; TNF-α, tumor necrosis factor α; HOMA-IR, homeostasis model assessment of insulin resistance; HOMA-β, homeostasis model assessment of β cell; HOMA-IS, homeostasis model assessment of insulin sensitivity; TG, triglyceride; TC, total cholesterol; LDL-C, low-density lipoprotein cholesterol; HDL-C, high-density lipoprotein cholesterol; TB, total bilirubin; DB, direct bilirubin; ALT, alanine aminotransferase; AST, aspartate aminotransferase; TBA, total bile acid; Scr, serum creatinine; eGFR, estimated glomerular filtration rate; HbA1c, glycosylated hemoglobin; FPG, fasting blood glucose; 2-h BG, 2-h blood glucose.

^#^ vs. prediabetes P < 0.05.

^##^ vs. prediabetes P < 0.01.

^*^ vs. healthy P < 0.05.

^**^ vs. healthy P < 0.01.

### Characteristics of Subgroups Divided by HOMA-IR

**Figure 2:**
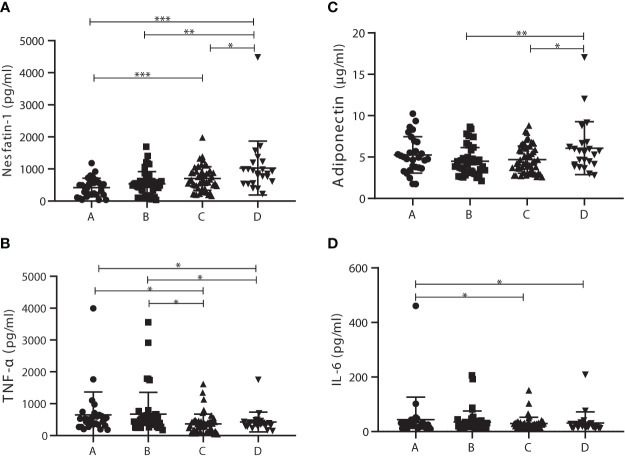
Comparison of serum levels of adiponectin, interleukin 6 (IL-6), tumor necrosis factor α (TNF-α), and nesfatin-1 among the homeostasis model assessment of insulin resistance (HOMA-IR) subgroups. **(A)** Nesfatin-1 levels in subgroup D were significantly increased compared with those in the other subgroups; nesfatin-1 levels in C subgroup were also significantly elevated in comparison to A subgroup. **(B)** TNF-α levels in subgroups A and B were outstandingly upregulated compared to those in subgroup C or D. **(C)** Adiponectin levels in D subgroup obviously increased compared with those in B or C subgroup. **(D)** IL-6 levels in A subgroup were significantly higher than those in C or D subgroup. * vs. Prediabetes with HOMA-IR under 2.8 *P* < 0.05. ns, non significantly.

To further investigate the roles of nesfatin-1, adiponectin, IL-6, and TNF-α in the development of T2DM *via* prediabetes, patients were distributed to four subgroups with cutoff 2.8 of HOMA-IR (29), which included 29 T2DM patients with HOMA-IR <2.8 (subgroup A), 43 T2DM patients with HOMA-IR >2.8 (subgroup B), 52 prediabetics with HOMA-IR <2.8 (subgroup C), and 23 prediabetics with HOMA-IR >2.8 (subgroup D). Characteristics of these subgroups were listed in **Supplement Table 1**. Nesfatin-1 levels in subgroup D (1,028.15 ± 839.70 pg/ml) were significantly increased compared with those in subgroup A (420.80 ± 289.70 pg/ml), B (536.41 ± 381.48 pg/ml), or C (702.81 ± 366.59 pg/ml). Meanwhile, nesfatin-1 serum levels in subgroup C outstandingly increased compared to those in A subgroup (**Figure 2A**). TNF-α levels in subgroups A (648.53 ± 719.06 pg/ml) and B (674.35 ± 683.62 pg/ml) were outstandingly upregulated compared to those in subgroups C (364.19 ± 308.69 pg/ml) and D (424.92 ± 311.17 pg/ml) (**Figure 2B**). Adiponectin in subgroup D (6.08 ± 3.20 µg/ml) was significantly higher than that in subgroup B (4.48 ± 1.66 µg/ml) or C (4.70 ± 1.65 µg/ml) but no difference between A (5.25 ± 2.20 µg/ml) and D (**Figure 2C**). IL-6 levels in A (43.92 ± 22.23 pg/ml) subgroup were obviously elevated in comparison to that in C (29.02 ± 19.02 pg/ml) or D (31.21 ± 14.95 pg/ml) subgroup except B (34.78 ± 14.38 pg/ml) subgroup (**Figure 2D**).

### Correlations Between Adiponectin and Other Indexes in Prediabetes

To further investigate the relationships among adiponectin, nesfatin-1, IL-6, and TNF-α in prediabetes, correlations between adiponectin and other indexes were analyzed, and the results were shown in **Table 2**. As described, serum levels of adiponectin were significantly correlated to IL-6 (R = 0.3918, *P* = 0.0005), TNF-α (R = 0.2939, *P* = 0.0105), insulin (R = -0.3068, *P* = -0.0074), HbA1c (R = -0.3404, *P* = 0.0048), HOMA-IR (R = 0.2904, *P* = 0.0115), and HOMA-β (R = 0.3354, *P* = 0.0033), whereas the correlation between adiponectin and nesfatin-1 was slight (R = -0.0146, *P* = 0.901).

**Table 2:** Correlations between adiponectin and other indexes.

Parameters	IL-6	TNF-α	Insulin	HbA1c	HOMA-IR	HOMA-β
Total participants						
R	0.1777	0.05	0.001	-0.1297	-0.062	0.060
*P* (two-tailed)	0.0084	0.46	0.987	0.080	0.356	0.373
Significant	**					
T2DM						
R	0.2597	0.068	-0.2198	0.012	0.2023	0.101
*P* (two-tailed)	0.0276	0.57	0.0636	0.923	0.088	0.3995
Significant	*					
Prediabetes						
R	0.3918	0.2939	-0.3068	-0.3404	0.2904	0.3354
*P* (two-tailed)	0.0005	0.0105	0.0074	0.0048	0.0115	0.0033
Significant	***	*	***	**	*	**
Healthy						
R	0.036	0.058	-0.2790	0.0117	-0.1961	0.3037
*P* (two-tailed)	0.7635	0.6282	0.0176	0.9393	0.0988	0.0095
Significant			*			**

T2DM, type 2 diabetes mellitus; IL-6, interleukin 6; TNF-α, tumor necrosis factor α; HOMA-IR, homeostasis model assessment of insulin resistance; HOMA-β, homeostasis model assessment of β cell; HbA1c, glycosylated hemoglobin.

*P < 0.05, **P < 0.01, ***P < 0.001.

### Correlations Between Adiponectin and Other Indexes Among Subgroups Divided by HOMA-IR

Adiponectin levels in the subgroups of prediabetes patients with HOMA-IR >2.8 were found to be strongly correlated with IL-6 (R = 0.6663, *P* = 0.005) or TNF-α (R = 0.7732, *P* < 0.001), but in the other three subgroups, there was no such correlation (**Table 3**). Moreover, serum adiponectin in the subgroup of prediabetics with HOMA >2.8 has powerful relevance to HDL-C (R = 0.6891, *P* = 0.0003), HDL-C/LDL-C (R = 0.7005, *P* = 0.0002), or TG (R = -0.4263, *P* = 0.0425). In T2DM with HOMA-IR >2.8 subgroup, such association was also presented between adiponectin and HDL-C: (R = 0.3591, *P* = 0.0195) or HDL-C/LDL-C (R = 0.3327, *P* = 0.0313). Additionally, serum adiponectin in the subgroup of prediabetics with HOMA-IR <2.8 was found to be significantly related to TG (R = -0.3016, *P* = 0.0298), LDL-C (R = -0.3359, *P* = 0.0149), or HDL-C/LDL-C (R = 0.3813, *P* = 0.0058).

**Table 3:** Correlation of adiponectin with other indexes of subgroups.

Parameters	IL-6	TNF-α	TG	LDL-C	HDL-C	HDL-C/LDL-C
T2DM HOMA-IR <2.8						
R	0.4069	0.2902	-0.1225	-0.0810	0.1192	0.1803
*P* (two-tailed)	0.0285	0.1267	0.5267	0.6761	0.5379	0.3493
Significant	*					
T2DM HOMA-IR>2.8						
R	-0.0162	-0.1271	0.0787	-0.1050	0.3591	0.3327
*P* (two-tailed)	0.9177	0.4167	0.6205	0.5083	0.0195	0.0313
Significant					*	*
Prediabetes HOMA-IR<2.8						
R	-0.0008	-0.1074	-0.3016	-0.3359	0.1718	0.3813
*P* (two-tailed)	0.9957	0.4486	0.0298	0.0149	0.2232	0.0058
Significant			*	*		**
Prediabetes HOMA-IR>2.8						
R	0.6663	0.7732	-0.4263	-0.1837	0.6891	0.7005
*P* (two-tailed)	0.0005	<0.0001	0.0425	0.4013	0.0003	0.0002
Significant	***	***	*		***	***

T2DM, type 2 diabetes mellitus; IL-6, interleukin 6; TNF-α, tumor necrosis factor α; TG, triglyceride; LDL-C, low-density lipoprotein cholesterol; HDL-C, high-density lipoprotein cholesterol; HOMA-IR, homeostasis model assessment of insulin resistance.

*P < 0.05, **P < 0.01, ***P < 0.001.

### Factors Involved in the Development of Prediabetes Into Type 2 Diabetes Mellitus

Risk factors that may affect prediabetes toward T2DM were assessed by logistic regression. Suffering from T2DM was introduced as the dependent variable, while the independent variables were IL-6, TNF-α, adiponectin, nesfatin-1, HOMA-IR, TG, TC, LDL-C, HDL-C, HDL-C/LDL-C, TB, DB, TBA, eGFR, HbA1c, FBG, and 2-h BG. The outcome in **Table 4** disclosed that TNF-α (OR = 5.541, *P* = 0.001), HOMA-IR (OR = 8.168, *P* < 0.001), TG (OR = 3.277, *P* = 0.024), eGFR (OR = 13.655, *P* = 0.027), and 2-h BG (OR = 56.802, *P* = 0.002) were the risk factors that may contribute to the development of prediabetes into T2DM, whereas insulin (OR = 0.157, *P* = 0.016), TC (OR = 0.325, *P* = 0.022), and TB (OR = 0.110, *P* = 0.012) were protective factors that may impede the process.

**Table 4:** Influence factors involved in the process from prediabetes to T2DM.

Variable	B	S_b_	Waldχ^2^	*P*	OR	95% CI
TNF-α	1.712	0.505	11.517	0.001	5.541	2.061~14.894
Insulin	-1.849	0.766	5.834	0.016	0.157	0.035~0.706
HOMA-IR	2.100	0.555	14.323	0.000	8.168	2.753~24.238
TG	1.187	0.526	5.087	0.024	3.277	1.168~9.190
TC	-1.124	0.491	5.241	0.022	0.325	0.124~0.851
TB	-2.211	0.878	6.346	0.012	0.110	0.020~0.612
DB	1.256	0.692	3.295	0.069	3.511	0.905~13.621
eGFR	2.614	1.182	4.889	0.027	13.655	1.346~138.55
2-h BG	4.040	1.299	9.674	0.002	56.802	4.455~724.282
Constant	-5.740	1.518	14.305	0.000	0.003	/

T2DM, type 2 diabetes mellitus; TNF-α, tumor necrosis factor α; HOMA-IR, homeostasis model assessment of insulin resistance; TG, triglyceride; TC, total cholesterol; TB, total bilirubin; DB, direct bilirubin; eGFR, estimated glomerular filtration rate; 2-h BG, 2-h blood glucose.

We further evaluated the influence factors of HOMA-IR and HbA1c by logistic regression. HOMA-IR and HbA1c were respectively set as dependent variables, and IL-6, TNF-α, adiponectin, nesfatin-1, TG, TC, LDL-C, HDL-C, HDL-C/LDL-C, TB, DB, TBA, eGFR, FBG, and 2-h BG were input as independent variables. As exhibited in **Supplement Table 2**, declined serum nesfatin-1 levels increased the risk of HOMA-IR (OR = 2.812, *P* = 0.010), while high FBG (OR = 4.082, *P* = 0.006) and HbA1c (OR = 2.523, *P* = 0.078) levels improved the risk of HOMA-IR. Elevated ALT (OR = 0.151, *P* = 0.018) could protect the body from HOMA-IR. In addition, downregulated adiponectin (OR = 6.238, *P* = 0.019) and elevated TB (OR = 12.382, *P* = 0.041), FBG (OR = 9.839, *P* < 0.001), and 2-h BG (OR = 3.454, *P* = 0.027) could positively connect to HbA1c, but escalated DB (OR = 0.168, *P* = 0.047) levels seemed to be a negative factor for HbA1c (**Supplement Table 3**).

## Discussion

In this study, we firstly revealed the differences of adiponectin, nesfatin-1, IL-6, and TNF-α between prediabetes and T2DM or healthy individuals in Chinese population. Our results disclosed that adiponectin levels in prediabetes were distinctly lower than those in the healthy, while no significant difference was observed when compared to those in T2DM, which was consistent with the consequences of previous studies (30, 31). Our data also proved that nesfatin-1 levels in prediabetes were obviously higher than those in T2DM but no significant difference when compared to those in the healthy, which is the first delineation, despite the fact that others demonstrated mildly lower nesfatin-1 levels in impaired fasting glycemia and impaired glucose tolerance in contrast to those in the healthy group (21). As for IL-6, our study detected remarkably increased IL-6 levels in prediabetes in contrast to those in healthy and that levels insignificantly dropped when compared to those in T2DM. Upadhyaya et al. (31) found that serum IL-6 levels in impaired fasting glucose group were dramatically higher than those in the normoglycemic group while mildly lower than those in the hyperglycemic group. Based on the experimental evidence that TNF-α concentration in prediabetes was significantly lower than that in T2DM but mildly higher than that in the healthy, Lainampetch et al. (32) concluded that TNF-α could promote prediabetes evolving into T2DM.

Prediabetes affects more than one-third of people in the adult population (33). If efficient intervention is not taken, prediabetes often develops into T2DM with an upward risk of cardiovascular disease (34). A few studies pointed out that prediabetes facilitates an over-inflammatory status that could negatively affect the cardiac performance in subjects with preserved ejection fraction and could make acute myocardial infarction condition far more serious (35, 36). Elevated oxidative stress markers such as nitrotyrosine, inflammatory cells, and cytokines were higher in prediabetes patients compared to those in normal glucose participants (37). Clinical and experimental evidence has shown that oxidative/nitrosative stress and inflammation associated with metabolic disorders such as obesity, hypertension, and diabetes conduce to left ventricular hypertrophy, fibrosis, diastolic dysfunction, heart failure, and ischemia/reperfusion damage (38–40). Metformin as a classical hypoglycemic drug that could downregulate serum glucose level by activating adenosine 5′-monophosphate activated protein kinase (AMPK) signaling pathway (41). A randomized controlled trial declared that metformin could significantly decrease the incidence of left anterior descending coronary endothelial dysfunction in prediabetes patients treated with metformin compared with those who did not take medication (37); other studies also confirmed metformin’s effects on reducing the risk of major adverse cardiac events (MACEs) in prediabetes (42, 43). The results of a 1-year follow-up study on obesity patients with prediabetes indicated that metformin could obviously reduce the levels of inflammation markers, such as IL-6, C Reactive Protein (CRP), TNF-α, and nitrotyrosine, as well as inflammation-correlated microRNAs (miRs) such as miR 195 and miR 27 (44). Insulin resistance and β-cell dysfunction occur very often in prediabetes patients. Obesity now is considered as one of the most important influence factors of inflammation and prediabetes (45, 46). Obesity, especially abdominal fat and pericoronary fat, showed higher inflammation markers, lifted glucose and insulin resistance levels, and elevated risk of developing MACE (36, 47). Some literature concluded that metformin could alleviate the inflammation burden in patients with pericoronary fat or abdominal fat and could restrain the expression of signaling factors such as nuclear factor kappa light-chain enhancer of activated B cells, peroxisome proliferator-activated receptor gamma, and sterol regulatory element-binding transcription factor 1 (36, 47). The above results indicate that metformin treatment may improve metabolic homeostasis and lower the risk of MACE in prediabetes patients.

Adiponectin is an important peptide secreted by adipocytes, which exhibits anti-diabetic, anti-inflammatory, and anti-atherogenic effects, and also functions as an insulin sensitizer through its action on the hypothalamus (48–50). A recent study revealed that adiponectin was a protective factor against ischemic heart disease (IHD) progression also in normal glucose tolerance (NGT) subjects undergoing percutaneous coronary intervention (PCI) (51). In our study, adiponectin in prediabetes was found to be significantly decreased compared with the healthy participants and positively correlated with TNF-α (R = 0.2939, *P* = 0.0105) or IL-6 (R = 0.3918, *P* = 0.0005), and their relationship was notably enhanced with HOMA-IR (TNF-α: R = 0.7732, *P* < 0.0001; IL-6: R = 0.6663, *P* = 0.0005). Taking together, it seems that decreased serum adiponectin levels were obviously correlated with elevated serum TNF-α or IL-6 levels. The explanation for this paradox may be that adiponectin plays a dual role of anti-inflammation and pro-inflammation in disease process. On one hand, adiponectin is a kind of anti-inflammation adipocytokine, and macrophage is the primary target of its anti-inflammation effect (52). Adiponectin inhibits the differentiation of myeloid progenitor cells, regulates the function of macrophage, and decreases the expression of Toll-like receptor 4 (53). Moreover, adiponectin suppresses the pro-inflammatory activities of M1 macrophage and promotes the activation and proliferation of M2 macrophage, as well as modulates macrophage polarization from M1 phenotype toward M2 cells (54, 55). On the other hand, it is worth noting that adiponectin appears to be an intensifying factor of pro-inflammatory reactions. Adiponectin induces human macrophage and T-cell differentiation to pro-inflammatory phenotype that resembles M1 other than M2 (56), and in human adipose tissue and placenta, adiponectin stimulates the release of TNF-α and IL-6 through Nuclear Factor-kappa B (NF-κB) and Extracellularly Regulated Kinase (ERK) signaling (57). Collectively, adiponectin functions as an anti-inflammatory cytokine or pro-inflammatory molecule by inhibiting the polarization of macrophage M1 (56) or eliciting the secretion of TNF-α and IL-6 in human adipose tissues (58). It can be inferred that adiponectin participates in the progression of prediabetes toward diabetes and changes its role of being anti-inflammatory or pro-inflammatory in response to different disease conditions.

Our results implicated that a falling nesfatin-1 level was a risk factor for HOMA-IR rising (OR = 2.812, *P* = 0.01), while reduced adiponectin level raised the risk of HbA1c elevation (OR = 6.238, *P* = 0.019). Studies focusing on aspects of nesfatin-1 and insulin resistance revealed that serum nesfatin-1 levels were decreased in insulin resistance-associated polycystic ovarian syndrome (59) and non-alcoholic fatty liver disease (60). An *in vivo* study demonstrated that injecting nesfatin-1 into the third cerebral ventricle of high-fat diet-fed rats contributed to promoting insulin-induced glucose uptake and insulin signaling through upregulating the phosphorylation levels of insulin receptors (20). Additionally, nesfatin-1 was observed to be augmented in gastric cancer and exerts a decent role in diagnosing gastric cancer (Area Under the Curve (AUC) = 0.857, 95% CI 0.769–0.946) with a threshold of 1.075 ng/ml (61). Although there is a growing body of evidence on nesfatin-1’s versatility, its receptor remains unknown.

In summary, this research revealed significant variations of adiponectin, nesfatin-1, IL-6, and TNF-α levels in the healthy, prediabetes, and T2DM, suggesting a significant but slow and gradual change during the progression from a healthy condition toward diabetes *via* prediabetes. We also stated the dual roles of adiponectin in the development of prediabetes into diabetes.

This study also has some limitations. Firstly, the research included only Chinese subjects, thus racial differences should be noticed when applying the conclusion to other populations. Secondly, the size of the participants in this study is relatively small, and more cases are required to verify the variations of adiponectin, nesfatin-1, TNF-α, and IL-6 in prediabetes and diabetes, as well as to make the conclusion more convincing. Thirdly, the cutoff HOMA-IR value of Chinese adults is vacant, and further studies are needed to define the exact value. Fourthly, a follow-up study should be conducted and focused on the variations of adiponectin, nesfatin-1, IL-6, and TNF-α in prediabetes patients with medical therapy such as metformin or other antidiabetic drugs, which may be helpful to illustrate the roles of these factors in the development of prediabetes as well as for early diagnosis and intervention of the disease. Last but not least, our study did not present the waist/hip ratio in the cohorts, which would reflect whether a person is abdominally obese or not; in this regard, a follow-up study is worth carrying out to explore the variation and function of adiponectin, inflammation, and oxidative stress markers in prediabetes with or without abdominal obesity. More importantly, the mechanisms of how adiponectin, nesfatin-1, TNF-α, and IL-6 are involved in the progression of prediabetes toward diabetes are worth our in-depth exploration.

## Data Availability Statement

The original contributions presented in the study are included in the article/**Supplementary Material**. Further inquiries can be directed to the corresponding author.

## Ethics Statement

The studies involving human participants were reviewed and approved by the Ethics Committee of Xiangya Hospital of Central South University. The patients/participants provided their written informed consent to participate in this study.

## Author Contributions

KH wrote the main article text. YL and YM prepared **Figures 1** and **2**. JW and HL prepared **Tables 1**–**4**. BY amended the first draft and approved the final version. All authors reviewed the article. All authors contributed to the article and approved the submitted version.

## Funding

This research received a grant from the Natural Science Foundation of Hunan Province (No. 1035-15).

## Conflict of Interest

The authors declare that the research was conducted in the absence of any commercial or financial relationships that could be construed as a potential conflict of interest.

## Publisher’s Note

All claims expressed in this article are solely those of the authors and do not necessarily represent those of their affiliated organizations, or those of the publisher, the editors and the reviewers. Any product that may be evaluated in this article, or claim that may be made by its manufacturer, is not guaranteed or endorsed by the publisher.

## Abbreviations

2-h BG, 2-h blood glucose; ALT, alanine aminotransferase; AST, aspartate aminotransferase; DB, direct bilirubin; eGFR, estimated glomerular filtration rate; ELISA, enzyme-linked immunosorbent assay; FBG, fasting blood glucose; HbA1c, hemoglobin A1c; HDL-C, high-density lipoprotein cholesterol; HOMA-IR, homeostasis model assessment of insulin resistance; HOMA-IS, homeostasis model assessment of insulin sensitivity; HOMA-β, homeostasis model assessment of beta-cell function; IL-6, interleukin 6; LDL-C, low-density lipoprotein cholesterol; Scr, serum creatinine; T2DM, type 2 diabetes mellitus; TB, total bilirubin; TBA, total bile acid; TC, total cholesterol; TG, total triglyceride; TNF-α, tumor necrosis factor α.
