# Supplementary material for: The Variation and Correlation of Serum Adiponectin, Nesfatin-1, IL-6, and TNF-α Levels in Prediabetes
Source: Front Endocrinol (Lausanne). 2022 Mar 3;13:774272. doi: 10.3389/fendo.2022.774272 (PMC8928772; doi:10.3389/fendo.2022.774272)
Supplement: Supplementary file 1 [file DataSheet_1.docx]

| Parameter | A | B | C | D |
| --- | --- | --- | --- | --- |
| Male/female | 20/9 | 30/13 | 34/18 | 15/8 |
| Age (years) | 57.90±10.27 | 58.19±9.24 | 58.02±9.38 | 59.78±10.58 |
| BMI (Kg/m2) | 22.14±1.79 | 23.40±4.72 | 22.07±2.35 | 22.42±3.37 |
| IL-6 (pg/ml) | 43.92±22.32^*#^ | 34.78±14.38 | 29.02±19.02 | 31.21±14.95 |
| TNF-α (pg/ml) | 648.53±719.06^**##^ | 674.35±683.62^**##^ | 364.19±308.69 | 424.92±311.17 |
| Nesfatin-1 (pg/ml) | 420.80±289.70^**###^ | 536.41±381.48^##^ | 702.81±366.59 | 1028.15±839.70^*^ |
| Adiponectin (µg/ml) | 5.25±2.20 | 4.48±1.66^#^ | 4.70±1.65^#^ | 6.08±2.13 |
| Insulin (µU/ml) | 5.07±2.00^#^ | 16.93±10.44^*^ | 5.69±2.13 | 23.46±16.01^*^ |
| HOMA-IR | 1.78±0.67^#^ | 6.93±6.42^*^ | 1.61±0.62 | 6.77±4.65^*^ |
| HOMA-β | 32.18±30.03^#^ | 88.90±93.10^*#^ | 40.31±16.31 | 158.11±107.89^*^ |
| HOMA-IS | 0.80±0.95^#^ | 0.20±0.09^*^ | 0.74±0.38 | 0.21±0.10^*^ |
| TG(µmol/L) | 2.59±4.03 | 2.11±1.24 | 2.07±1.89 | 2.30±1.72 |
| TC (µmol/L) | 5.11±2.08 | 4.96±1.15 | 5.57±1.08 | 5.48±0.93 |
| LDL-C (mmol/L) | 3.17±1.00 | 3.25±1.00 | 3.53±0.73 | 3.44±0.68 |
| HDL-C (mmol/L) | 1.08±0.21^#^ | 1.19±0.37 | 1.25±0.27 | 1.35±0.36 |
| HDL/LDL | 0.37±0.11 | 0.46±0.62^*#^ | 0.18±0.38 | 0.19±0.05 |
| TB (µmol/L) | 10.50±4.70 | 12.03±5.71 | 11.35±5.91 | 13.11±6.45 |
| DB (µmol/L) | 5.37±2.38 | 5.73±2.95 | 5.50±2.78 | 6.34±2.96 |
| ALT (U/L) | 34.38±47.31 | 23.77±15.32 | 32.67±26.60 | 28.51±19.21 |
| AST (U/L) | 29.69±24.11 | 22.57±7.98 | 28.41±13.45 | 26.65±9.00 |
| TBA (µmol/L) | 5.31±5.50 | 4.85±4.91 | 4.55±4.11 | 4.80±2.99 |
| Scr (µmol/L) | 104.51±76.24 | 98.01±67.72 | 80.26±14.34 | 79.20±10.22 |
| eGFR (ml·min^-1^· (1.73 m^2^)^-1^) | 71.70±22.96 | 76.52±23.43 | 81.63±30.69 | 79.34±10.49 |
| HbA1c (%) | 7.49±1.68^*#^ | 8.03±2.08^*#^ | 5.98±0.25 | 5.88±0.27 |
| FPG (mmol/l) | 8.19±2.84^*#^ | 9.18±3.30^*#^ | 6.37±0.36 | 6.48±0.26 |
| 2hBG(mmol/l) | 14.73±4.63^*#^ | 14.93±5.91^*#^ | 9.21±1.31 | 8.68±1.48 |

**Supplement table 1 Anthropometric and Clinical characteristics of the study subjects in subgroups divided by HOMA-IR**

Abbreviations: A: T2DM with HOMA-IR<2.8; B: T2DM with HOMA-IR>2.8; C: Prediabetes with HOMA-IR<2.8; D: Prediabetes with HOMA-IR>2.8; BMI: body mass index;IL-6: interleukin 6;TNF-α: tumor necrosis factor α; HOMA-IR: homeostasis model assessment of insulin resistance; HOMA-β: homeostasis model assessment of β cell; HOMA-IS: homeostasis model assessment of insulin sensitivity; TG: triglyceride; TC: total cholestenone; LDL-C: low density lipoprotein-cholesterol; HDL-C: high density lipoprotein-cholesterol; TB: total bilirubin; DB: direct bilirubin; ALT: alanine aminotransferase; AST: aspartate aminotransferase; TBA: total bile acid; Scr: serum creatinine; eGFR: estimated glomerular filtration; HbA1c: glycosylated hemoglobin; FPG: fasting blood glucose; 2hBG: 2 hours blood glucose. *: vs Prediabetes with HOMA-IR under 2.8 P<0.05, **: vs Prediabetes with HOMA-IR under 2.8 P<0.01; #: vs Prediabetes with HOMA-IR above 2.8 P<0.05, ##: vs Prediabetes with HOMA-IR above 2.8 P<0.05.

**Supplement table 2 Influence factors of elevated HOMA-IR**

| Variable | B | S_b_ | Waldχ^2^ | *P* | OR | 95%CI |
| --- | --- | --- | --- | --- | --- | --- |
| Nesfatin-1 | 1.034 | 0.404 | 6.565 | 0.010 | 2.812 | 1.275~6.201 |
| ALT | -1.893 | 0.797 | 5.638 | 0.018 | 0.151 | 0.032~0.719 |
| FBG | 1.407 | 0.507 | 7.697 | 0.006 | 4.082 | 1.511~11.028 |
| HbA1c | 0.926 | 0.526 | 3.099 | 0.078 | 2.523 | 0.900~7.072 |
| Constant | -3.082 | 0.531 | 33.641 | 0.000 | 0.046 | / |

**Supplement table 3 Influence factors of elevated HbA1c**

| Variable | B | S_b_ | Waldχ^2^ | *P* | OR | 95%CI |
| --- | --- | --- | --- | --- | --- | --- |
| Adiponectin | 1.831 | 0.779 | 5.521 | 0.019 | 6.238 | 1.355~28.717 |
| HDL-C | 1.517 | 0.628 | 5.830 | 0.016 | 4.557 | 1.330~15.605 |
| TB | 2.516 | 1.231 | 4.180 | 0.041 | 12.382 | 1.110~138.140 |
| DB | -1.785 | 0.898 | 3.950 | 0.047 | 0.168 | 0.029~0.976 |
| TBA | 2.980 | 1.822 | 2.675 | 0.102 | 19.681 | 0.554~699.551 |
| FBG | 2.286 | 0.552 | 17.132 | 0.000 | 9.839 | 3.332~29.052 |
| 2hBG | 1.239 | 0.561 | 4.886 | 0.027 | 3.454 | 1.151~10.365 |
| IL-6 | 0.815 | 0.476 | 2.933 | 0.087 | 2.259 | 0.889~5.739 |
| TC | 0.935 | 0.498 | 3.526 | 0.060 | 2.548 | 0.960~6.756 |
| Constant | -2.459 | 0.478 | 26.419 | 0.000 | 0.085 | / |
